# Experimental Study on Mechanical and Microstructural Properties of Foam Concrete Incorporating Desert Sand as Partial Fine Aggregate

**DOI:** 10.3390/ma19112269

**Published:** 2026-05-27

**Authors:** Aihemaitijiang Tuerhong, Qingguang Zeng, Nueraili Maimaitituersun, Shihai Gui, Zuojun Ning, Erxing Peng

**Affiliations:** 1 School of Transportation, Kashi University, Kashi 844000, China; ai005@ksu.edu.cn (A.T.); 13018357084@163.com (Q.Z.); nurali2023@ksu.edu.cn (N.M.); erxingpeng@lzb.ac.cn (E.P.); 2 School of Civil and Hydraulic Engineering, Huazhong University of Science and Technology, Wuhan 430074, China; shihai_gui@hust.edu.cn

**Keywords:** desert sand foam concrete, compressive strength, pore structure, deep learning-based image analysis, macroscopic and microscopic relationship

## Abstract

The escalating depletion of river sand resources poses a critical sustainability challenge for the production of foam concrete, while the reinforcement mechanism of locally abundant aeolian sand in cementitious matrices remains insufficiently quantified. To address this gap, the present study investigates the feasibility of partially substituting river sand with Taklamakan desert sand at replacement ratios of 0%, 20%, and 40%, under varying water-to-binder (W/B) ratios (0.3, 0.4, 0.5) and sand-to-binder (S/B) ratios (0, 0.3, 0.6). To correlate macroscopic performance with microstructural features, compressive strength was tested, and pore structure evolution was characterized using deep learning-based image segmentation, supplemented by XRD and SEM analyses. Results indicate that increasing the W/B ratio from 0.3 to 0.5 elevates porosity by up to 111.7%, resulting in a 47.4% reduction in compressive strength. Similarly, raising the S/B ratio from 0 to 0.6 introduces additional interfacial transition zones (ITZs) and dilutes the cementitious phase, which consequently weakens the matrix and leads to a strength reduction of up to 66.5%. However, the contribution of desert sand replacement exhibits a pronounced “S/B ratio dependence”. Notably, at an S/B ratio of 0.6 and a 40% desert sand replacement rate, the compressive strength experiences a significant increase of 51.4% compared to the control group. Quantitative analysis further reveals that the compressive strength follows positive and negative power-law relationships with dry density and porosity, respectively. Ecological assessment shows that desert sand foam concrete (DSFC) with high S/B and high desert sand replacement ratio reduces embodied CO_2_ by 36.4% and cost by 26.9% compared to conventional foam concrete. These findings demonstrate that partial replacement of river sand by desert sand offers a low-carbon, cost-effective solution for foam concrete.

## 1. Introduction

Foam concrete is a lightweight cellular material widely employed in engineering owing to its integrated structural, thermal, and acoustic performance [1]. Its compressive strength—governed by the intricate coupling between the solid matrix strength and the void geometry—typically follows a power-law relationship with density [2]. Unlike dense concrete, foam concrete predominantly fails through the crushing of thin pore walls; consequently, refining the pore structure to mitigate stress concentrations (Griffith flaws) constitutes the cornerstone of its mechanical enhancement [2,3]. A narrow distribution of small, uniform, spherical pores yields markedly higher strength, whereas large, irregular voids act as fracture initiators [3]. Achieving such an optimal pore structure is highly sensitive to mix-proportion parameters. In foam concrete, an excessively low water-to-binder ratio (W/B < 0.3) produces a stiff paste that exerts high shear forces during mixing, rupturing the delicate foam bubbles and degrading the pore network—a phenomenon counter-intuitive to that of conventional dense concrete [4]. Liu et al. [4] identified an optimal W/B window of 0.4–0.6, within which the rheology is balanced sufficiently to encapsulate the bubbles without rupturing them. Concurrently, the sand-to-binder ratio (S/B) governs the rigid skeleton within the cell walls: while an increased sand content suppresses shrinkage, an excessive S/B dilutes the cementitious binder. At high S/B ratios, the strength becomes dominated by aggregate interlock, rendering particle morphology critical—coarse, angular sand tends to puncture fragile bubbles, whereas fine, rounded sand integrates as a micro-filler rather than a disruptive inclusion [5].

Despite these optimization strategies, conventional foam concrete still relies heavily on cement and river sand, thereby exacerbating resource depletion and carbon emissions [6]. Driven by a threefold increase in global material extraction over the past forty years (from 22 billion tons in 1970 to 70 billion tons in 2010), the building sector now accounts for 39% of global carbon emissions, while 28% of this is operational carbon, 11% is embodied carbon generated during material manufacturing and construction. To mitigate this impact, the World Green Building Council has established ambitious targets: a 40% reduction in embodied carbon by 2030 and net-zero operational carbon by 2050 [7]. Realizing these goals heavily relies on the development of green building materials that significantly cut upfront carbon emissions. Although industrial by-products such as ground granulated blast-furnace slag and silica fume have been employed to refine the pore structure through chemical or physical filling effects, their limited regional availability and reactive chemical nature constrain their large-scale, scalable application [8,9,10]. Aeolian sand, an ultra-fine (0.075–0.25 mm), well-graded, and rounded material formed by long-term wind erosion, offers a highly viable and abundant alternative whose modification mechanism is dominated by physical packing rather than chemical reactivity.

Existing literature demonstrates that the incorporation of aeolian sand enhances the initial flowability of fresh foam concrete via a “ball-bearing” lubrication mechanism. Beyond this rheological improvement, the structural benefits become particularly pronounced at an optimal replacement level. The fine particles function as micro-fillers, optimizing the pore size distribution by increasing the proportion of beneficial micropores while mitigating the formation of harmful macropores. Consequently, these synergistic effects significantly densify the cementitious matrix [11]. However, exceeding this threshold induces pore coalescence and a pronounced strength degradation, manifesting a clear “S/B ratio dependence” [11]. Existing investigations on aeolian sand foam concrete (ASFC), nevertheless, predominantly rely on traditional single-factor analysis and lack a systematic, multi-parameter optimization framework that simultaneously incorporates macroscopic mechanical performance, microscopic pore-structure refinement, and life-cycle environmental impact. Bridging this research gap requires a precise quantification of the macro–micro relationship. Traditional techniques for characterizing the pore structure, such as mercury intrusion porosimetry (MIP) and X-ray computed tomography (XCT), are often hindered by high costs, low throughput, and limited equipment accessibility. By contrast, deep learning-based image analysis—particularly the U-Net architecture for semantic segmentation—has emerged as a transformative tool [12]. It enables the rapid and cost-effective classification of “pore” versus “matrix” pixels, allowing for the instantaneous extraction of critical statistical descriptors including the equivalent diameter, circularity (shape factor), and fractal dimension (boundary complexity) [3].

The Taklamakan Desert in Xinjiang, China, holds vast reserves of aeolian sand characterized by high sphericity and chemical stability (predominantly composed of quartz). Exploiting this local resource not only alleviates pressure on river ecosystems but also substantially reduces the carbon footprint associated with long-distance transportation [13,14].

In view of the above, this study systematically investigates the development of desert sand foam concrete (hereinafter abbreviated as DSFC) through the partial substitution of Taklamakan aeolian sand for coarse sand, aiming to enhance the compressive strength while reducing the cement consumption. To this end, four primary objectives are pursued: (1) to elucidate the coupled effects of the water-to-binder ratio, sand-to-binder ratio, and desert sand replacement rate on the flowability and macroscopic compressive strength; (2) to analyze the evolution of the hydration products and phase composition; (3) to quantitatively characterize the porosity and pore size distribution by means of deep learning-based image analysis; and (4) to establish quantitative macro–micro correlations to guide the optimization of mix designs. By mapping these correlations, the present work seeks to position aeolian sand not merely as an inert filler but as an active performance enhancer in low-carbon construction materials.

## 2. Specimen Preparation and Test Methods

### 2.1. Preparation of Test Specimens

#### 2.1.1. Materials

**Figure 1:**
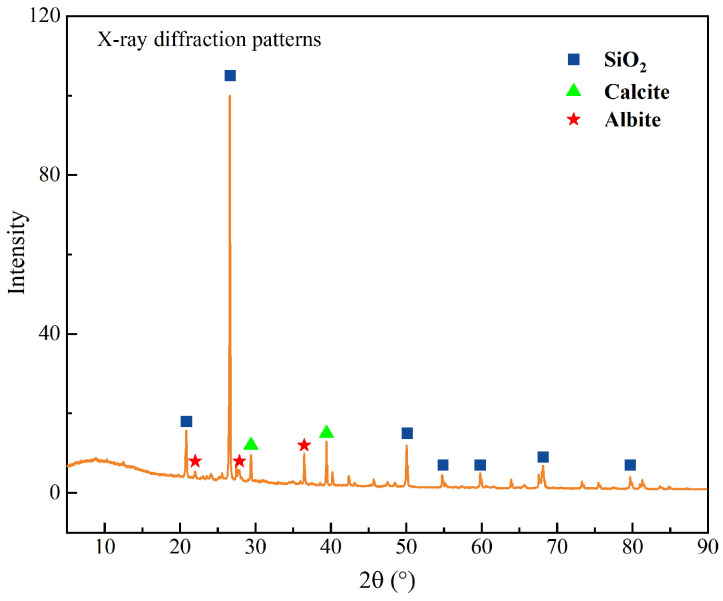
X-ray diffraction pattern of desert sand.

**Figure 2:**
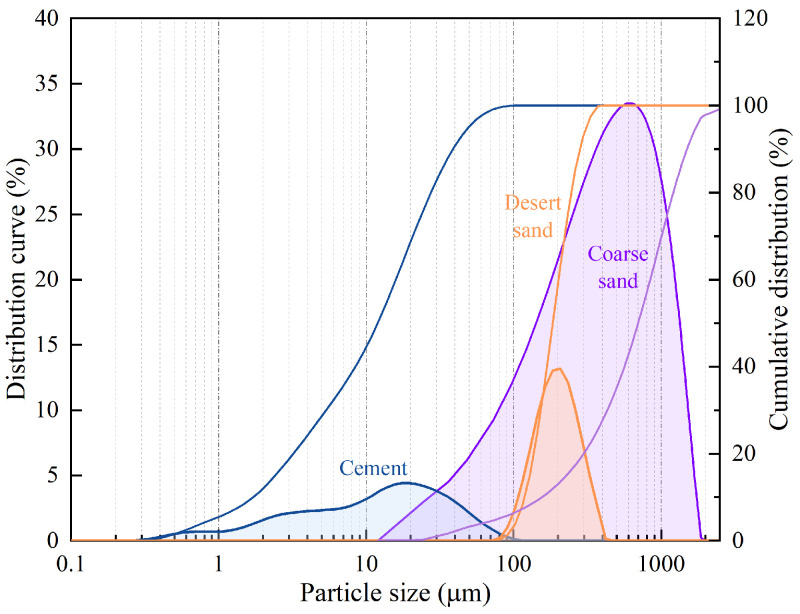
Particle size distribution curves of cement, coarse sand, and desert sand.

The raw materials used in this study comprised cement, coarse sand, desert sand, foaming agent, foam stabilizer, and accelerating admixture. The cement was P·O 42.5 ordinary Portland cement (OPC) supplied by the Kashgar Tianshan Cement factory; its chemical composition is listed in Table 1. To eliminate the influence of impurities commonly present in river sand, standard sand with a SiO_2_ content greater than 98.5% and physical properties conforming to GB/T 17671-2021 [15] was used as the coarse aggregate. The desert sand was sampled from the Taklamakan Desert near Maigaiti County in the Kashgar region, and its chemical composition is presented in Table 2. The X-ray diffraction patterns of the desert sand are shown in Figure 1. The particle size distributions of the above materials are illustrated in Figure 2. A high-efficiency composite foaming agent with a solid content of 50%, supplied by LEIMO Chemical, was adopted as the foaming agent, and calcium stearate (CaS) was incorporated as the foam stabilizer. Calcium silicate hydrate served as the accelerating admixture, the principal role of which is to induce the rapid nucleation and growth of C-S-H gel within the slurry, thereby accelerating hydration and suppressing plastic shrinkage and settlement of DSFC. The densities of all the aforementioned materials are summarized in Table 3.

#### 2.1.2. Mix Design

The target dry density of the desert sand foam concrete in this study was set at 1600 kg/m^3^. The principal variables considered were the water-to-binder ratio, sand-to-binder ratio, and desert sand replacement rate, with the aim of investigating their coupled effects on the mechanical properties and microstructure of foam concrete. Specifically, the water-to-binder ratio was set to 0.3, 0.4, and 0.5; the sand-to-binder ratio was set to 0, 0.3, and 0.6; and the substitution rate of desert sand for coarse sand was set to 0%, 20%, and 40%, respectively. The mix designs were formulated in accordance with the Technical Specification for Application of Foam Concrete (JGJ/T 341-2014) [16] (Equations (1) and (2)), and the proportions of each constituent are listed in Table 4. The mass ratio of foaming agent to water in the foaming solution was 1:30, the foam stabilizer dosage was 1/10 of the foaming agent mass, and the early-strength agent was added at 1.0% of the cement mass.

(1) $$\rho_{d}=S_{a}\left(m_{c}+m_{s}\right)$$

(2) $$V_{foam}=K\left(1-\frac{m_{c}}{\rho_{c}}-\frac{m_{s}}{\rho_{s}}-\frac{m_{w}}{\rho_{w}}\right)$$

In the Equations (1) and (2), $S_{a}$ is the mass coefficient of foam concrete, taken as 1.2 in accordance with the specification, and $\rho_{d}$ represents the design dry density of the foam concrete, in kg/m^3^; $m_{c}$, $m_{s}$, $m_{w}$ denote the masses of the cementitious material, aggregate, and water per cubic meter of foam concrete in kg, respectively; $\rho_{c}$, $\rho_{s}$, and $\rho_{w}$ are the corresponding densities of cement, aggregate, and water, in kg/m^3^; $V_{\mathrm{foam}}$ represents the foam volume, in L; and *K* is the safety factor, taken as 1.05 in this study according to the specification.

#### 2.1.3. Preparation of Specimens

**Figure 3:**
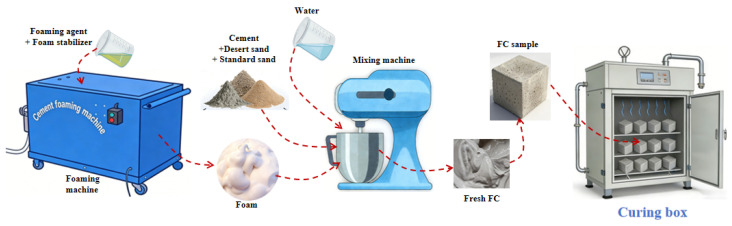
Preparation procedure of foam concrete (FC).

**Figure 4:**
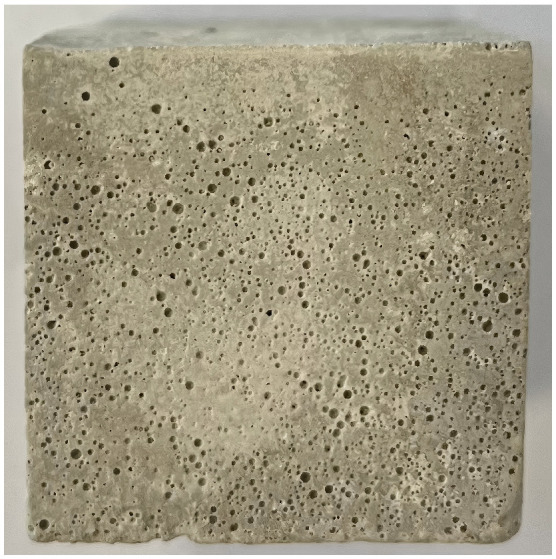
Foam concrete sample.

The detailed preparation procedure of desert sand foam concrete was as follows: (1) the cement and sand were stirred in a mixer at 60 r/min for 60 s; (2) a mixture of water and accelerating admixture was added to the mixer and stirred at 120 r/min for 120 s to obtain a uniform cement mortar; (3) the foaming agent and foam stabilizer were combined and introduced into a foaming machine, where foam was generated by the physical foaming method; (4) the resulting foam (density: 40 kg/m^3^) was added to the mortar and mixed at 60 r/min for an additional 30 s; and (5) the fresh mixture was cast into 40 mm × 40 mm × 40 mm cubic molds (3 specimens per group). The specimens were cured at room temperature for 24 h, then demolded and transferred to a standard curing chamber maintained at 20 ± 2 °C and a relative humidity greater than 95% for 28 days. The complete preparation flow is depicted in Figure 3. The prepared foam concrete specimens are shown in Figure 4.

### 2.2. Test Methods

#### 2.2.1. Flowability and Mechanical Properties

The flowability and dry density were determined according to the test method specified in the Technical Specification for Application of Foam Concrete (JGJ/T 341-2014) [16]. In accordance with the Chinese standard “Test Methods for Foamed Concrete and Products” (GB/T 43487-2023) [17], the compressive strength of the specimens at 7, 14, and 28 days was determined using an LCY-300B universal testing machine (Zhejiang Lice Instrument Equipment Co., Ltd., Shaoxing, China) with a maximum load capacity of 300 kN. A constant loading rate of 2.5 kN/s was applied to all specimens, and the average value of three parallel specimens was taken as the final compressive strength to ensure the reliability of the results.

#### 2.2.2. Pore Structure Analysis

In this study, deep learning-based image processing was employed to perform a quantitative analysis of the pore structure of the foam concrete. The procedure was as follows. First, high-resolution digital images of the polished cross-sections of the specimens were acquired with a high-definition digital camera and uniformly cropped to representative regions of 1024 × 1024 pixels for subsequent analysis. The cross-sectional images were then segmented using the SAM model embedded in ImageJ software 1.54 to achieve a precise identification of the pore regions. Compared with conventional grayscale-threshold segmentation, this deep learning-based approach effectively overcomes the misclassification induced by the grayscale gradients caused by optical shadows along pore edges, thereby preventing a single pore from being erroneously identified as multiple regions. The segmentation results were subsequently binarized to separate the pores from the matrix, generating black-and-white binary images suitable for quantitative analysis. Finally, characteristic parameters such as porosity and pore size distribution were extracted from the binary images using the built-in analytical tools of ImageJ.

#### 2.2.3. Microstructural Analysis

(1) XRD

The phase composition of DSFC was determined using a TD-3500 X-ray diffractometer manufactured by Dandong Tongda Science & Technology Co., Ltd., Dandong, China The scans were performed in the 2θ range of 5°–80° at a step size of 0.02°/s and a scanning speed of 2.4°/min, with a tube voltage of 30 kV and a tube current of 20 mA.

(2) SEM

The microstructural features of DSFC were observed with a Phenom Prox fully automated scanning electron microscope (Phenom-World B.V., Eindhoven, The Netherlands), which provides a secondary electron resolution of 5.0 nm at an accelerating voltage of 15 kV. The sample preparation prior to SEM observation involved the following steps: a section with a flat fracture surface was first selected, after which the surrounding material was trimmed and polished to a final size of 3 mm × 3 mm × 2 mm; the prepared sample was then immersed in anhydrous ethanol for 24 h, dried with an electric blower, and finally sputter-coated with gold to enhance its conductivity, thereby ensuring the testing stability and image quality.

## 3. Results and Discussion

### 3.1. Flowability

**Figure 5:**
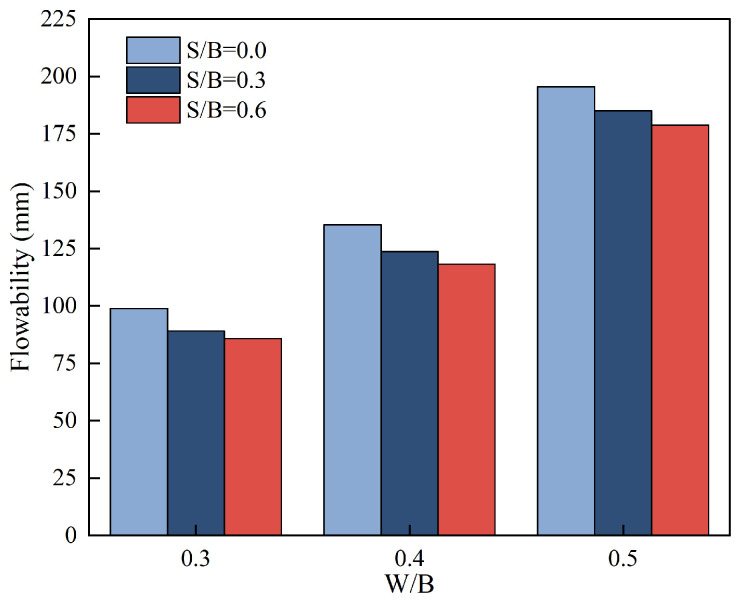
The effect of different water-to-binder (W/B) and sand-to-binder (S/B) ratios on the flowability of foam concrete.

To ensure adequate workability and self-compacting properties, the flowability of fresh foam concrete must be carefully controlled. Figure 5 illustrates the variation in the flowability of foam concrete with different sand-to-binder ratios as the water-to-binder ratio changes, in the absence of desert sand. As the water-to-binder ratio increases from 0.3 to 0.5, the flowability of the foam concrete improves significantly. This is primarily attributable to the increased free water content induced by the higher water-to-binder ratio, which effectively decreases the apparent viscosity of the slurry and thereby enhances its flow performance. Notably, only at a water-to-binder ratio of 0.5 does the flowability fall within the range of 160–200 mm specified in the Technical Specification for Application of Foam Concrete (JGJ/T 341-2014) [16]; in the absence of water-reducing admixtures, the slurries corresponding to the other water-to-binder ratios fail to satisfy this requirement. Furthermore, at a fixed water-to-binder ratio, the flowability gradually decreases as the sand-to-binder ratio increases from 0.0 to 0.6. This is because the surface of standard sand particles is relatively rough, allowing them to adsorb part of the free water; concurrently, the inter-particle friction and the packing effect of the sand grains increase the internal flow resistance of the slurry, thereby reducing its flowability. The higher the sand-to-binder ratio, the greater the amount of free water adsorbed and the greater the internal resistance, leading to a more pronounced reduction in flow performance.

**Figure 6:**
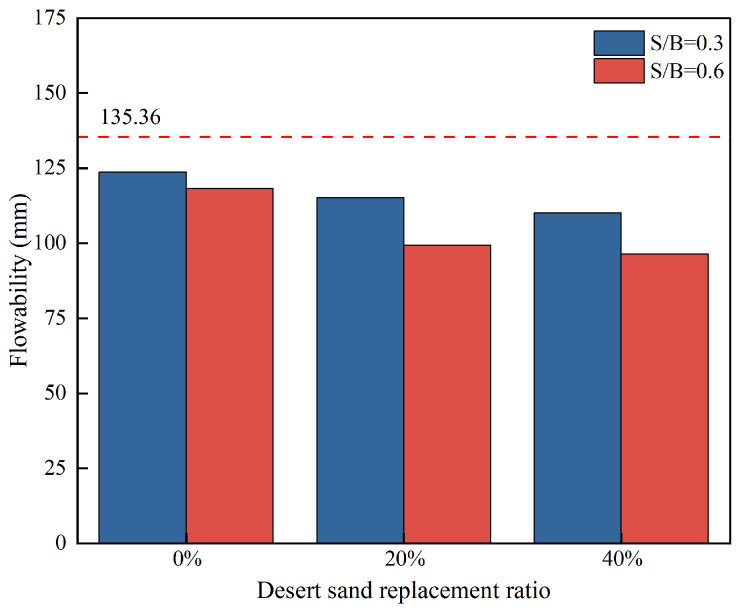
The relationship between desert sand replacement rate and flowability.

Figure 6 shows the variation in the flowability of foam concrete with different sand-to-binder ratios (0.3 and 0.6) as a function of the desert sand replacement rate (0%, 20%, and 40%) at a fixed water-to-binder ratio of 0.4. The dashed line in the figure denotes the reference flowability (135.36 mm) corresponding to a sand-to-binder ratio of 0. The results indicate that the flowability further decreases monotonically as the desert sand replacement rate increases. This phenomenon is mainly attributable to the particle characteristics of desert sand: its particles are finer than those of standard sand, possess relatively smooth surfaces, and exhibit a larger specific surface area. Consequently, during mixing, desert sand adsorbs more water, thereby reducing the amount of free water available to lubricate the slurry. At the same time, the packing effect of the fine particles increases the internal flow resistance of the slurry, jointly aggravating the loss of flowability. The higher the desert sand replacement rate, the more pronounced the adsorption and packing effects, and consequently the more significant the deterioration in workability.

### 3.2. Compressive Strength

**Figure 7:**
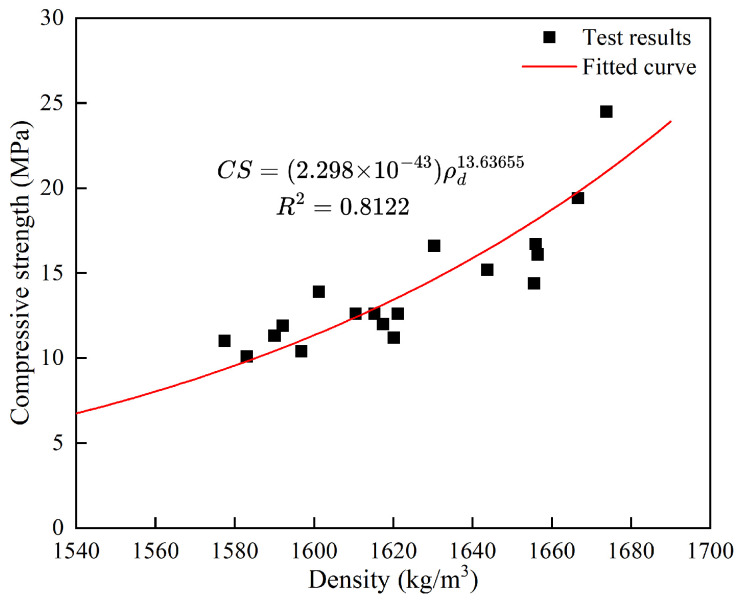
The relationship between compressive strength and density. Note: $\rho_{d}$ is dry density and CS is compressive strength after 28 days.

The dry densities of the test specimens ranged from 1570 to 1670 kg/m^3^. Figure 7 illustrates the relationship between the compressive strength and dry density of DSFC. The results indicate that the compressive strength exhibits a significant positive power-law relationship with dry density. Specifically, when the dry density increased from 1580.1 kg/m^3^ to 1656.5 kg/m^3^, the compressive strength rose from 10.1 MPa to 16.1 MPa, corresponding to an increase of 59.4%. This trend is highly consistent with the conclusions of previous studies—namely, that a higher density of foam concrete is generally accompanied by a higher compressive strength [18]—indicating that a denser internal structure typically enhances the load-bearing capacity of the foam concrete.

**Figure 8:**
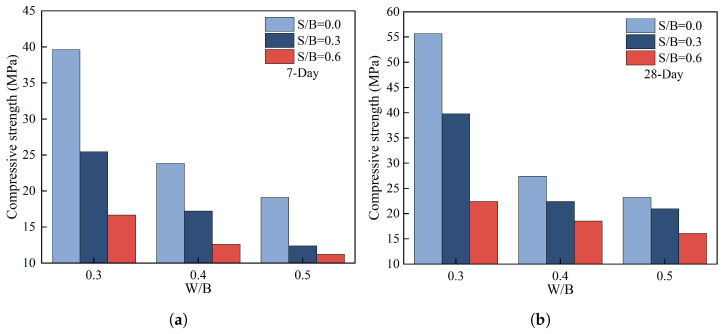
The relationship between the compressive strength of foam concrete and the water-to-binder (W/B) at different sand-to-binder (S/B) ratios. (**a**) 7-day curing. (**b**) 28-day curing.

Figure 8 illustrates the variation in the compressive strength of foam concrete with different sand-to-binder ratios as a function of the water-to-binder ratio in the absence of desert sand. As shown in Figure 8a, the compressive strength of the foam concrete decreases with increasing water-to-binder ratio across all curing ages. Figure 8b further reveals that, at a sand-to-binder ratio (S/B) of 0.3, the compressive strength drops from 39.8 MPa to 20.9 MPa as the water-to-binder ratio increases from 0.3 to 0.5, corresponding to a decline of 47.4%. This decrease is mainly because a higher water-to-binder ratio increases the free water content in the cement paste, leading to the formation of more residual pores upon hardening, which in turn reduces the matrix density and strength [19]. At a fixed water-to-binder ratio, the compressive strength generally decreases with an increasing sand-to-binder ratio, with the maximum reduction reaching 66.5%. This phenomenon is attributable to the existence of an interfacial transition zone (ITZ) between the coarse sand particles and the cement paste; this region constitutes a structural weak point owing to local water accumulation and an uneven distribution of hydration products [20]. The higher the sand-to-binder ratio, the larger the proportion of sand particles in the paste and the greater the total area of the ITZ [21], thereby introducing a greater number of microscopic defects [22]. Concurrently, a high sand-to-binder ratio reduces the relative content of the cement paste, leading to a corresponding decrease in the relative content of hydration products, weakened bonding between sand particles, and ultimately a reduction in the overall compressive strength. With prolonged curing, the compressive strength of all mix proportions improves significantly. Even under the unfavorable combination of S/B = 0.6 and W/B = 0.5, the 28-day compressive strength (16.1 MPa) is 43.6% higher than the corresponding 7-day value (11.2 MPa). This indicates that, with increasing curing time, the continued hydration of cement particles produces more C-S-H gel and Portlandite, which progressively fill the pores within the paste, increasing the microstructural density and thereby gradually enhancing the compressive load-bearing capacity of the material.

**Figure 9:**
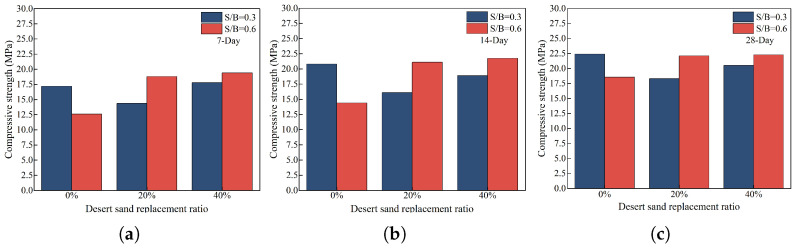
The relationship between the compressive strength of foam concrete and the replacement rate of desert sand at different sand-to-binder (S/B) ratios. (**a**) 7-day curing. (**b**) 14-day curing. (**c**) 28-day curing.

Figure 9 shows the variation in the compressive strength of foam concrete with different sand-to-binder ratios (0.3 and 0.6) as a function of the desert sand replacement rate (0%, 20%, and 40%) at a fixed water-to-binder ratio of 0.4. The results reveal that the influence of the desert sand replacement rate on strength is closely correlated with the sand-to-binder ratio. For instance, at S/B = 0.3, the compressive strength first decreases and then increases as the desert sand replacement rate increases. As shown in Figure 9a, when the desert sand replacement rate increases from 0% to 20%, the compressive strength decreases from 17.2 MPa to 14.4 MPa, a reduction of 16.5%; when the replacement rate further reaches 40%, the strength recovers to 17.8 MPa, slightly exceeding that of the control group. At S/B = 0.6, the compressive strength exhibits a positive correlation with the desert sand replacement rate. As shown in Figure 9b, as the replacement rate increases from 0% to 40%, the compressive strength rises from 14.4 MPa to 21.8 MPa, corresponding to an increase of 51.4%; similarly, in Figure 9c, the strength increases from 18.6 MPa to 22.3 MPa, an increase of 19.9%.

The above strength variation patterns can be attributed to the combined effects of the particle packing effect and the interfacial transition zone (ITZ) effect of desert sand [23]. (1) Under low sand-to-binder ratio conditions (S/B = 0.3), the cement matrix is relatively abundant. When the desert sand replacement rate is low (e.g., 20%), its high specific surface area adsorbs a substantial amount of free water, decreasing the slurry workability and inhibiting cement hydration, which in turn introduces additional interfacial defects; consequently, the compressive strength of the specimens exhibits a downward trend. However, when the desert sand replacement rate is increased to a moderate level (e.g., 40%), the filling effect of the fine particles becomes increasingly pronounced, refining the microstructure of the foam concrete and densifying the ITZ, thereby partially restoring the strength. (2) Under high sand-to-binder ratio conditions (S/B = 0.6), the framework formed by standard sand exhibits a high inherent porosity, and the addition of desert sand can therefore exert a more significant physical filling effect. As the replacement rate increases, the fine particles effectively occupy the voids between aggregates and promote the densification of the paste–aggregate interface. This filling-dominated mechanism markedly improves the microstructure and consequently enhances the strength. In summary, the role of desert sand in the composite system depends on the balance between its filling and interfacial effects, which is jointly governed by the sand-to-binder ratio and the replacement rate; the mechanisms by which it modulates the microstructure and macroscopic properties differ markedly under different mix proportions.

In addition, as the curing period was extended from 7 to 28 days, the compressive strength of all mix proportions improved significantly. Taking S/B = 0.6 as an example (corresponding to Figure 9a,c), at a replacement rate of 20% the strength increased from 18.8 MPa at 7 days to 22.1 MPa at 28 days, corresponding to an increase of 17.6%. This indicates that the introduction of desert sand does not significantly inhibit the late-stage hydration of the cementitious system.

### 3.3. Pore Structure Analysis

**Figure 10:**
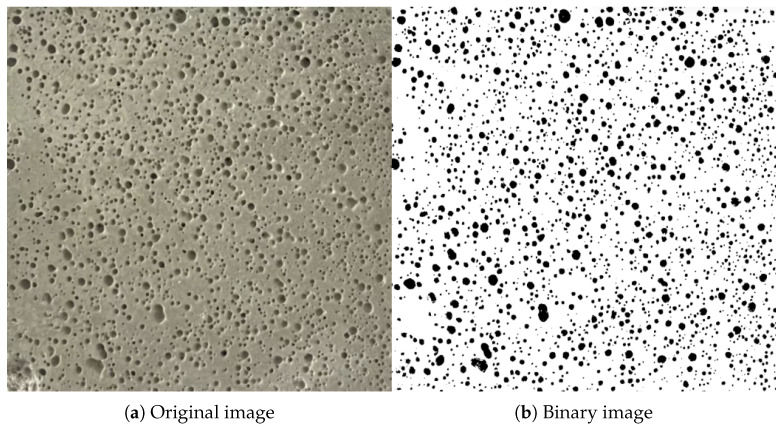
Results of deep learning-based image segmentation.

To elucidate the variation patterns of the pore structure of foam concrete with the water-to-binder ratio, sand-to-binder ratio, and desert sand replacement rate, Section 3.3.1 and Section 3.3.2 provide a detailed discussion of the evolution of porosity and pore size distribution under these variables, with the aim of providing a basis for the optimization of mix designs. The cross-sections of the specimens and the corresponding binarized images are shown in Figure 10.

#### 3.3.1. Porosity

**Figure 11:**
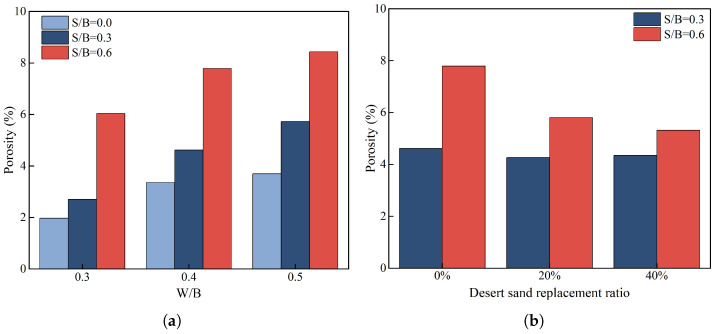
Porosity of foam concrete at different sand-to-binder (S/B) ratios. (**a**) Porosity versus water-to-binder (W/B) ratios. (**b**) Porosity versus desert sand replacement ratio.

Porosity is one of the key microstructural characteristics of foam concrete that warrants particular attention. Figure 11a illustrates the variation in porosity of foam concrete with different sand-to-binder ratios (without desert sand) as a function of the water-to-binder ratio. As shown in the figure, under all three sand-to-binder ratio conditions, the porosity of the foam concrete increases with the water-to-binder ratio, with the maximum increase reaching 111.7%. This phenomenon can be attributed to two factors: (1) an increased water-to-binder ratio implies a higher free water content in the slurry, the evaporation of which during curing generates additional capillary pores and directly raises the matrix porosity [24]; and (2) an increased water-to-binder ratio reduces the slurry viscosity and weakens the foam stability, rendering the bubbles susceptible to coalescence and rupture, which results in a higher proportion of large-volume pores and a further increase in the total porosity [24]. At a fixed water-to-binder ratio, the porosity also increases markedly with the sand-to-binder ratio. For example, at W/B = 0.4, the porosity increases from 3.4% to 7.8% as S/B increases from 0.0 to 0.6. This is because, with an increased sand-to-binder ratio, a greater proportion of cement is replaced by coarse sand, reducing the volume fraction of the cementitious material so that the voids between sand particles cannot be sufficiently filled. Moreover, the ITZ between the sand and the paste becomes more porous owing to the insufficient hydration products, leading to a significant rise in the overall porosity. In summary, an increased water-to-binder ratio and a reduction in the cementitious content jointly contribute to an elevated porosity in the foam concrete.

Figure 11b shows the variation in the porosity of foam concrete at a water-to-binder ratio of 0.4 and different sand-to-binder ratios (0.3 and 0.6) as a function of the desert sand replacement rate (0%, 20%, and 40%). Under low sand-to-binder ratio conditions (S/B = 0.3), as the desert sand replacement rate increased from 0% to 40%, the porosity exhibited a slight decrease followed by a slight rebound, dropping from 4.6% (at 0% replacement) to 4.4% (at 40% replacement)—a reduction of approximately 4.4%, indicating only minor variation. Under high sand-to-binder ratio conditions (S/B = 0.6), the influence of the desert sand replacement rate on porosity was much more pronounced: the porosity values were 7.8%, 5.8%, and 5.3% with increasing replacement rate, corresponding to a reduction of approximately 32.1%. These results indicate that the effect of replacing coarse sand with desert sand on the porosity of foam concrete is highly dependent on the sand-to-binder ratio: at a low sand-to-binder ratio (S/B = 0.3), the substitution effect is limited; whereas at a high sand-to-binder ratio (S/B = 0.6), the introduction of desert sand can significantly reduce the matrix porosity. This phenomenon may be explained as follows. Desert sand typically possesses a finer particle size distribution and higher sphericity, in sharp contrast to coarse sand, which is characterized by larger particles and angular edges [25]. In a system with a high sand-to-binder ratio (S/B = 0.6), sand particles occupy a large volume fraction, and the voids formed by coarse sand packing are relatively large; the fine desert sand particles can effectively fill these voids and form a denser particle packing structure, thereby significantly reducing the packing porosity of the matrix [11]. This filling effect becomes more prominent in systems with higher sand contents, which explains the more substantial decrease in porosity observed at S/B = 0.6. By contrast, in systems with a low sand-to-binder ratio (S/B = 0.3), the volume fraction of sand particles is small and the voids formed by coarse sand packing are inherently limited, leaving insufficient space for the desert sand to fill, so that its regulatory effect on the total porosity is correspondingly weaker. Overall, the reduction in porosity induced by the substitution of desert sand depends strongly on the sand-to-binder ratio, and the filling effect of fine aggregates plays a particularly significant role in systems with high sand-to-binder ratios.

#### 3.3.2. Pore Size Distribution

**Figure 12:**
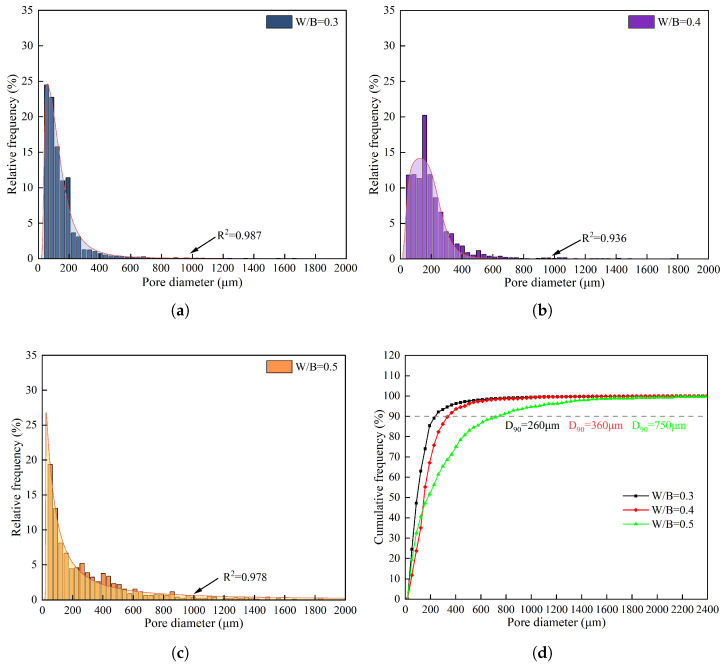
Pore size distribution of foam concrete with different water-to-binder (W/B) ratios. (**a**) Relative frequency distribution, W/B = 0.3. (**b**) Relative frequency distribution, W/B = 0.4. (**c**) Relative frequency distribution, W/B = 0.5. (**d**) Cumulative frequency distribution. Note: The red dotted line represents the fitted non-standard log-normal distribution curve. R^2^ is the coefficient of determination.

**Figure 13:**
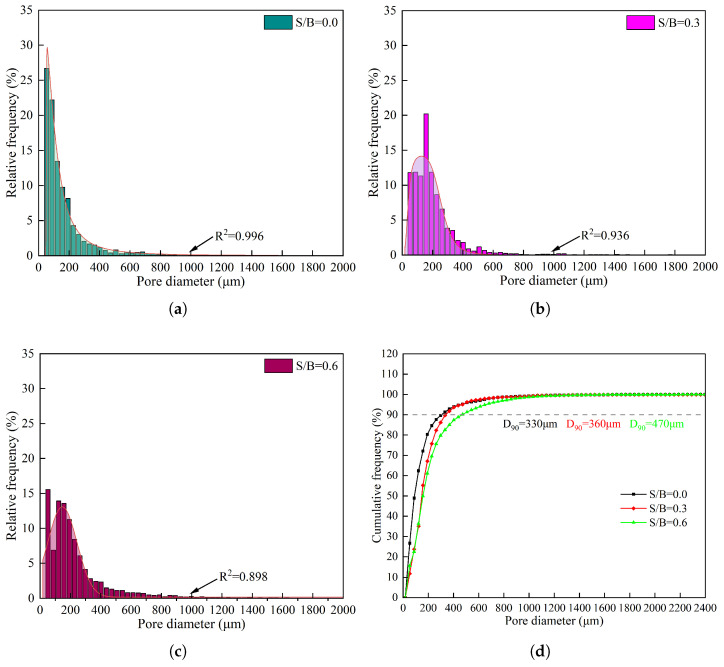
Pore size distribution of foam concrete with different sand-to-binder (S/B) ratios. (**a**) Relative frequency distribution, S/B = 0.0. (**b**) Relative frequency distribution, S/B = 0.3. (**c**) Relative frequency distribution, S/B = 0.6. (**d**) Cumulative frequency distribution. Note: The red dotted line represents the fitted non-standard log-normal distribution curve. R^2^ is the coefficient of determination.

The pore size distribution is one of the most influential microstructural characteristics governing the physical and mechanical properties of foam concrete. Figure 12 and Figure 13 present the pore size distribution and cumulative distribution curves of foam concrete at different water-to-binder and sand-to-binder ratios in the absence of desert sand. To quantitatively evaluate the variation in pore size, the value of D90—corresponding to the 90th percentile of the cumulative distribution curve—was selected as a key descriptor.

As illustrated by the pore size distribution curves in Figure 12a–c, the pore size distribution of foam concrete approximately follows a non-standard log-normal distribution, with the most frequent pore size lying in the range of 50 μm to 200 μm. As the water-to-binder ratio increases from 0.3 to 0.5, the proportion of pores larger than 200 μm rises from 10.9% to 40.7%, an increase of 270.2%. Furthermore, the corresponding D90 values at water-to-binder ratios of 0.3, 0.4, and 0.5 are 260 μm, 360 μm, and 750 μm, respectively, (Figure 12d), indicating that D90 also increases monotonically with the water-to-binder ratio. This implies that an elevated water-to-binder ratio leads to a significant increase in the proportion of large pores within the foam concrete. The phenomenon can be attributed to the fact that an increased water-to-binder ratio reduces the slurry viscosity and weakens its capacity to confine the foam, allowing the bubbles to coalesce and expand during mixing and casting, thereby forming larger pores and increasing the proportion of macropores.

Figure 13 shows the pore size distribution and cumulative distribution curves at different sand-to-binder ratios. As shown in the figure, when the sand-to-binder ratio increases from 0.0 to 0.6, the frequency of pores larger than 200 μm rises from 15.6% to 30.4%, corresponding to an increase of 95.3%. Furthermore, as shown in Figure 13d, the D90 values for specimens with sand-to-binder ratios of 0.0, 0.3, and 0.6 are 330 μm, 360 μm, and 470 μm, respectively, demonstrating that D90 increases monotonically with the sand-to-binder ratio. The above data indicate that an increased sand-to-binder ratio likewise leads to a higher proportion of large pores in foam concrete. This is because a higher sand-to-binder ratio reduces the relative amount of cementitious material, weakening the ability of the cement paste to encapsulate the bubbles and consequently promoting bubble coalescence and rupture, which in turn produces voids of larger diameter.

**Figure 14:**
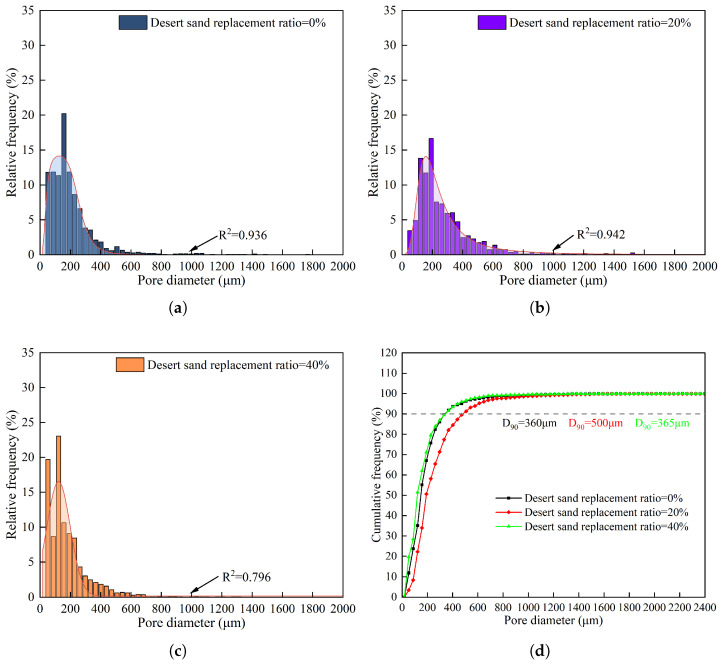
Pore size distribution at different desert sand replacement ratios (S/B = 0.3, W/B = 0.4). (**a**) Relative frequency distribution, 0% replacement. (**b**) Relative frequency distribution, 20% replacement. (**c**) Relative frequency distribution, 40% replacement. (**d**) Cumulative frequency distribution. Note: The red dotted line represents the fitted non-standard log-normal distribution curve. R^2^ is the coefficient of determination.

**Figure 15:**
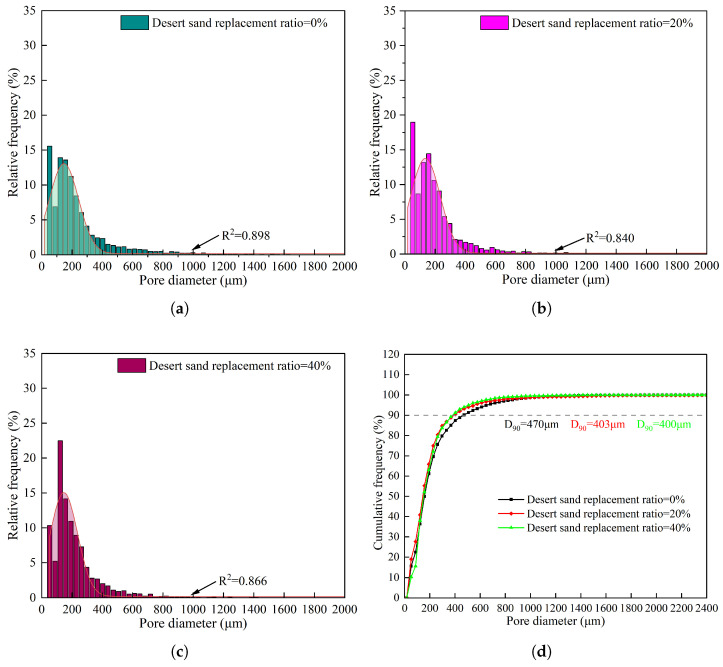
Pore size distribution at different desert sand replacement ratios (S/B = 0.6, W/B = 0.4). (**a**) Relative frequency distribution, 0% replacement. (**b**) Relative frequency distribution, 20% replacement. (**c**) Relative frequency distribution, 40% replacement. (**d**) Cumulative frequency distribution. Note: The red dotted line represents the fitted non-standard log-normal distribution curve. R^2^ is the coefficient of determination.

Figure 14 and Figure 15 show the variation in the pore size distribution of foam concrete with different sand-to-binder ratios (0.3 and 0.6) as a function of the desert sand replacement rate (0%, 20%, and 40%) at a fixed water-to-binder ratio of 0.4. The pore size distribution of foam concrete under these mix proportions also follows a non-standard log-normal distribution, with the most frequent pore size lying in the range of 50 μm to 300 μm.

At a sand-to-binder ratio (S/B) of 0.3, the proportion of pores larger than 300 μm exhibits an initial increase followed by a decrease as the desert sand replacement rate increases from 0% to 40%, with values of 13.9%, 28.7%, and 13.1%, respectively; the corresponding D90 values (Figure 14d) are 360 μm, 500 μm, and 365 μm, respectively, displaying the same trend. This is because, at a replacement rate of 20%, the fine desert sand—whose particle size is much smaller than that of coarse sand—introduces a large number of new interfaces, increasing the specific surface area of the slurry and producing a sharp rise in viscosity. This hinders the uniform dispersion of foam during mixing, reduces the inter-bubble spacing, and significantly increases the probability of bubble coalescence, thereby raising the proportion of large pores. Concurrently, insufficient hydration products in the newly formed desert sand–paste ITZ produce a loose pore structure, further amplifying the proportion of large pores and causing D90 to surge to 500 μm. At this stage, owing to the low replacement rate, the “interfacial effect” of desert sand dominates while its filling effect is not yet evident. When the replacement rate is further increased to 40%, the filling effect of desert sand as a fine aggregate becomes significantly enhanced: the small, rounded desert sand particles effectively fill the voids between sand grains and within the paste, optimizing the particle gradation and promoting the densification of the matrix.

At a sand-to-binder ratio (S/B) of 0.6, as the desert sand replacement rate increases, the proportion of pores larger than 300 μm is 20.3%, 15.7%, and 16.3%, respectively, exhibiting an initial decrease followed by stabilization; the corresponding D90 values are 470 μm, 400 μm, and 403 μm, respectively, also showing an initial decrease followed by an essentially constant trend, as illustrated in Figure 15. This phenomenon can be attributed to the fact that, under high sand-to-binder ratio conditions, the reference system itself suffers from insufficient paste, resulting in an incomplete initial pore structure. When the desert sand replacement rate reaches 20%, the fine desert sand effectively fills the voids left between sand particles owing to the lack of paste, optimizing the particle gradation and enhancing the ability of the paste to coat the aggregates. This reduces the compressive constraint exerted on the foam by the matrix, lowers the probability of bubble coalescence, and thereby suppresses the increase in the proportion of large pores, leading to a decrease in the frequency of pores larger than 300 μm. When the replacement rate is further increased to 40%, the continued addition of desert sand causes the particle bulk density to approach saturation, beyond which the filling effect can no longer be enhanced and its capacity to refine the pore structure approaches the upper limit of this system; meanwhile, owing to the limitation in the total slurry volume, no large-pore rebound caused by interfacial defects occurs, so that the frequency of pores larger than 300 μm and the D90 value both stabilize. In summary, the influence of desert sand on the pore size distribution of foam concrete varies with the sand-to-binder ratio and is essentially governed by the dynamic balance between its “interfacial effect” and “filling effect”.

### 3.4. Relationship Between Mechanical Properties and Microstructural Parameters

**Figure 16:**
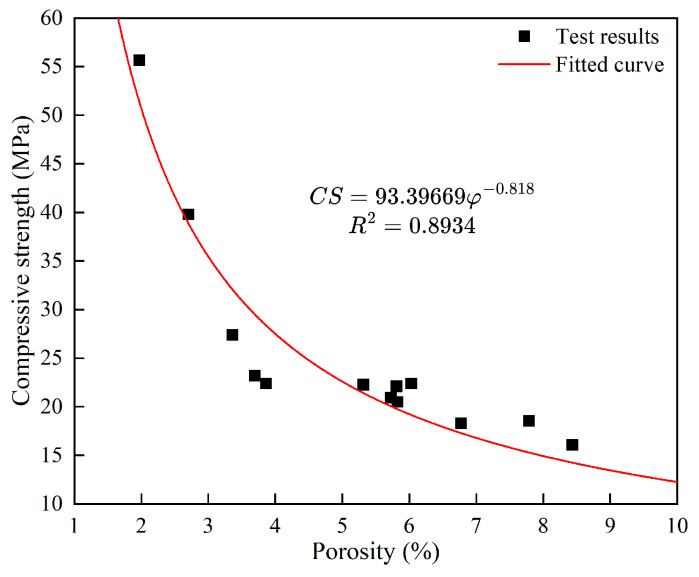
The relationship between porosity and compressive strength. Note: φ is porosity and CS is compressive strength after 28 days.

Porosity and pore size distribution, as critical microstructural characteristics of foam concrete, are key factors determining its mechanical properties. Figure 16 illustrates the correlation between the porosity and the compressive strength of the foam concrete, and the results reveal that the two follow a significant negative power-law decay relationship. Within the range of 2.0% to 8.4%, the compressive strength decreases rapidly with increasing porosity: as the porosity rises from 2.0% to 8.0%, the compressive strength drops sharply from 55.7 MPa to 16.1 MPa, a reduction of 71.1%, indicating that even a slight increase in porosity can substantially deteriorate the strength of the material. This is because pores constitute inherent defects within the matrix; a higher porosity reduces the proportion of solid matrix per unit volume and correspondingly diminishes the effective load-bearing cross-sectional area. Under external loading, stress concentrations readily develop around the pores, thereby inducing a reduction in the overall strength of the material [26].

**Figure 17:**
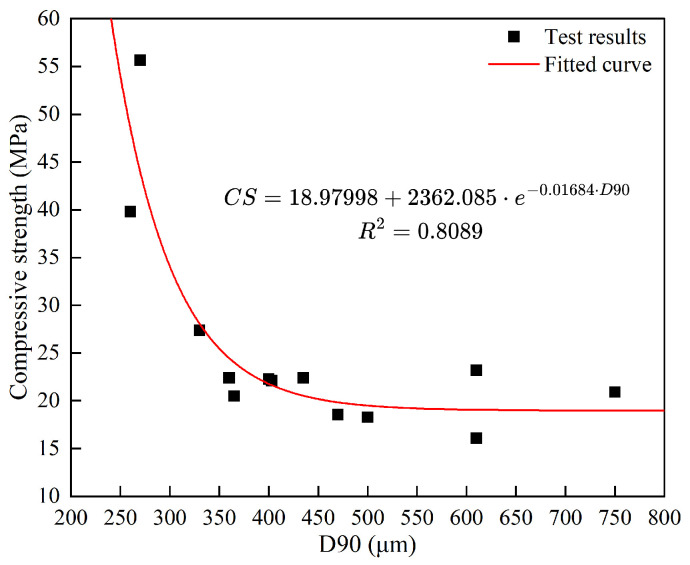
Relationship between D90 values and compressive strength. Note: CS is compressive strength after 28 days.

As a characteristic parameter describing the pore size distribution, D90 effectively reflects the uniformity of the pore structure and the proportion of large pores. Figure 17 illustrates the correlation between the D90 value and the compressive strength of the foam concrete. The data show that when D90 increases from 270 μm to 330 μm, the compressive strength decreases by 50.8%; within the range of 330–500 μm, the strength decreases by a further 33.2%; and as D90 continues to increase, the rate of strength decline tends to plateau. This phenomenon reveals a “saturation tendency” in the detrimental effect of large pores (>500 μm) on strength. Further analysis indicates that, within the fine pore range, even a slight increase in the proportion of coarse pores exerts a significant negative impact on the load-bearing capacity of the foam concrete; however, once the overall pore size coarsens beyond a certain extent, the lower bound of foam concrete strength becomes dominated by the inherent strength of the cement matrix, and the marginal weakening effect of further increases in pore size is correspondingly reduced. From a microscopic standpoint, an increase in D90 indicates a higher proportion of coarse pores in the system. Owing to the greater curvature of coarse pore walls, more pronounced stress concentration effects develop under compressive loading, thereby substantially reducing the critical load for crack initiation and ultimately weakening the load-bearing capacity of the material [27]. In summary, the macroscopic compressive strength of foam concrete is jointly governed by the total porosity and the proportion of large pores, and the weakening effect of the latter on strength exhibits distinct marginal saturation characteristics.

### 3.5. Microstructural Analysis

**Figure 18:**
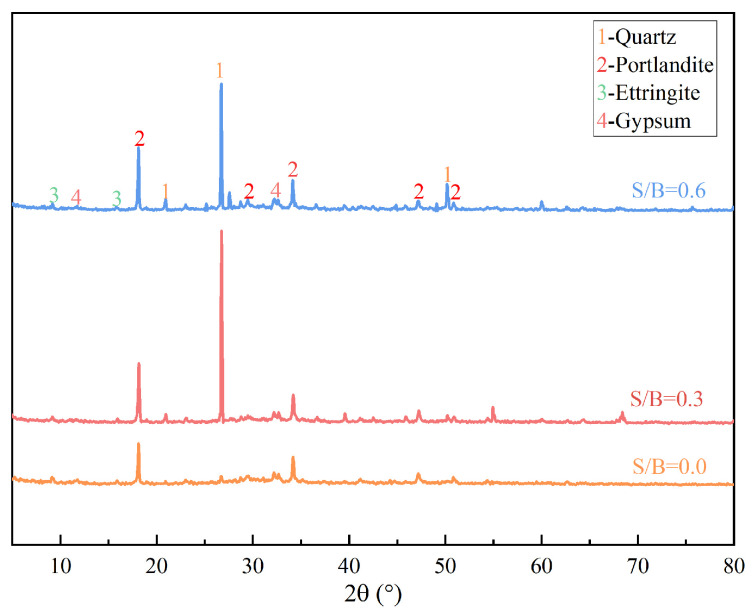
The hydration products of foam concrete (FC) with different S/B ratios.

In this study, XRD and SEM were employed to systematically characterize the composition of the hydration products in DSFC. The XRD analysis (Figure 18) reveals that the principal phases of the hydration products include portlandite, ettringite, gypsum, and magnesium aluminium hydroxide hydrate, accompanied by minor amounts of thaumasite and calcite. In addition, the characteristic diffraction peaks of SiO_2_ (quartz) are clearly visible, originating mainly from the standard sand and desert sand. A pronounced diffuse diffraction background is also observed in the 2θ range of 20°–50°, manifesting as an upward-curving “convex peak” that is a typical feature of an amorphous or weakly crystalline phase, confirming the presence of calcium silicate hydrate (C-S-H) or calcium aluminosilicate hydrate (C-A-S-H) gels. These gels constitute the primary cementing phase of the matrix; however, owing to their amorphous or weakly crystalline nature, no distinct crystalline peaks are visible in the XRD pattern.

**Figure 19:**
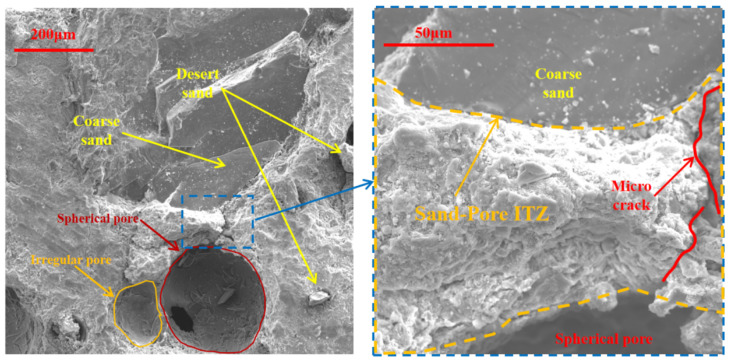
SEM images of the sand–pore ITZ of DSFC.

To further characterize the hydration products, SEM-EDS analysis was performed. Figure 19 presents the microstructure of the DSFC matrix, which is composed of cement hydration products, aggregates, and pores. Coarse sand and desert sand are non-pozzolanic and therefore do not participate in the hydration reaction; coarse sand mainly serves as a skeletal framework in the system, mitigating the shrinkage and settlement of the foam concrete, whereas desert sand particles are dispersed within the pores and gel of the matrix and thereby refine its microstructure. As further shown in the figure, the pores are enveloped by various hydration products, forming intact and independent pore wall structures. The pores exhibit two morphological forms—circular and elliptical—and the dark regions within the pores indicate internal damage. As observed in the close-up view (Figure 19), abundant and dense hydration products have formed between the coarse sand and the pores, as well as between adjacent pores, generating a reliable interfacial transition zone that constitutes the primary source of strength in the foam concrete. In addition, microcracks are observed within the matrix, which, together with the internal damage of the pores, form weak zones; under external loading these regions are prone to stress concentration, leading to premature failure of the material.

**Figure 20:**
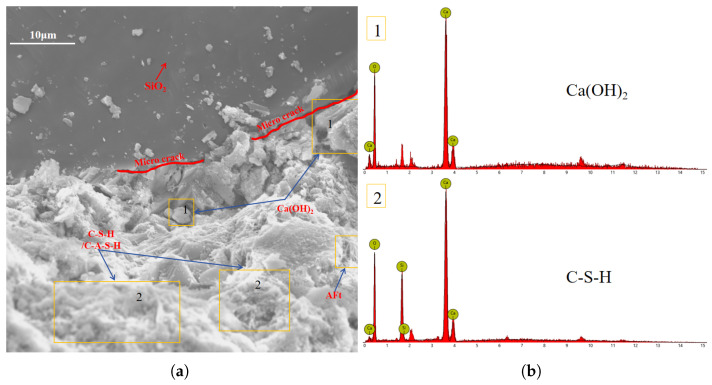
SEM-EDS results of DSFC hydration products. (**a**) SEM micrograph. (**b**) EDS spectra of the marked regions.

Figure 20 shows the principal phases of DSFC, in which large amounts of C-S-H/C-A-S-H gel and Ca(OH)_2_ crystals, together with a small quantity of acicular portlandite and other hydration products, can be clearly identified; these observations are consistent with the XRD findings. The Ca(OH)_2_ (portlandite) crystals predominantly grow in a layered morphology among the hydration products; however, their stacking structure is relatively loose and may introduce additional micropores. By contrast, the C-S-H or C-A-S-H gels markedly enhance the integrity of the pore walls and the matrix by effectively filling the pores and tightly encapsulating and bonding other solid-phase particles [28]. This mechanism contributes to the formation of a denser and more robust pore structure within the foam concrete, thereby positively enhancing both the macroscopic density and the mechanical properties. Ettringite crystals typically exhibit a needle-like morphology and, by interlocking with one another, form a relatively loose three-dimensional network structure; they belong to the early-stage hydration products.

**Figure 21:**
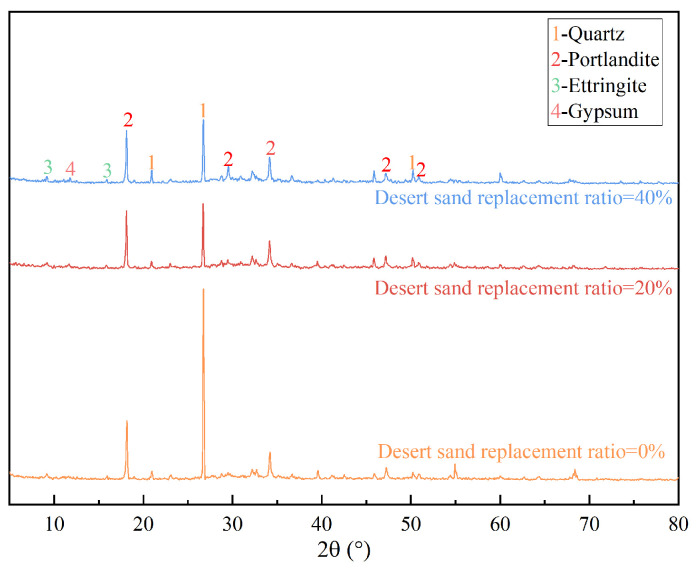
The hydration products of foam concrete (FC) with different desert sand content.

Figure 18 and Figure 21 present the XRD patterns of the specimens at different sand-to-binder ratios and desert sand replacement rates, respectively. As shown in Figure 18, with an increasing sand-to-binder ratio, the relative intensity of the diffraction peak corresponding to Ca(OH)_2_ increases significantly, while the diffuse peaks of ettringite and the amorphous gel phase relatively decrease. Combined with semi-quantitative analysis, it is evident that the relative content of Ca(OH)_2_ crystals in the system rises with the sand-to-binder ratio, whereas the formation of ettringite and C-S-H gel decreases accordingly. The increase in calcium hydroxide content—together with its weakly cementing layered structure—and the relative deficiency of C-S-H gel as the key cementing phase jointly lead to an increase in internal porosity and a decrease in structural density. These microstructural changes directly result in an increase in porosity with increasing sand-to-binder ratio (Figure 11a), which in turn leads to a reduction in the strength of the specimens. It can therefore be concluded that the deterioration of the microstructure is the fundamental cause of the decline in macroscopic compressive strength, an inference that is in good agreement with the mechanical test results.

Furthermore, the influence of the desert sand replacement rate was investigated in detail (Figure 21). At a fixed sand-to-binder ratio of 0.3, three sets of specimens with desert sand replacement rates of 0%, 20%, and 40% were compared. The phases with the highest diffraction intensities in the patterns remained SiO_2_ and Ca(OH)_2_. As an early-stage hydration product, the content of ettringite was already at a relatively low level in the later stages, contributing only marginally to the final strength. The semi-quantitative analysis of the principal phases reveals that, as the desert sand replacement rate increased from 0% to 20% and then 40%, the relative content of Ca(OH)_2_ first rose from 17.4% to 27.5% and then slightly decreased to 24.0%, displaying a pattern of initial increase followed by a slight decline; in contrast, the content of the C-S-H/C-A-S-H gel—the core cementing phase—exhibited a fluctuating trend, first decreasing by approximately 3% and then increasing by about 2%. It is precisely this evolution in the content of the key cementing phases at the microscopic scale that governs the filling of matrix pores and the overall density, thereby modulating the porosity (Figure 11b) and ultimately driving the corresponding variations in the macroscopic strength.

### 3.6. Analysis of Ecological Effects

Given the construction industry’s growing demand for sustainable materials, a systematic comparison of the life-cycle environmental impacts and economic viability between DSFC and conventional foam concrete is essential. As the carbon emissions and costs associated with raw materials are critical determinants of the practical application of concrete, the life cycle assessment (LCA) framework was employed to comprehensively quantify the embodied CO_2_ emissions and economic costs of the raw materials required to produce 1 m^3^ of DSFC and conventional FC. The total embodied CO_2_ emissions value was calculated using Equation (3).

(3) $$\mathrm{Embodied}\;{\mathrm{CO}}_{2}\;\mathrm{emission}=\sum \left(m_{i}\times {\mathrm{EF}}_{i}\right)$$

where *m_i_* denotes the mass of material *i*, and EF*_i_* represents the corresponding emission factor.

The embodied CO_2_ emission factor, embodied energy values, and unit costs of the raw materials [29,30,31,32] are summarized in Table 5. The unit cost of each material is computed based on local material costs and the prevailing currency exchange rate. The mix designs for FC2 and FC13 presented in Table 5 correspond to No. 2 and No. 13 in Table 4, respectively. In addition, the eco-strength efficiency corresponding to the 28-day compressive strength of both the DSFC and FC specimens was evaluated using Equation (4) provided in Ref. [33].

(4) $$ESE=\frac{EC}{CS}$$

where ESE denotes the eco-strength efficiency, CS is the 28-day compressive strength obtained in this study (in MPa), and EC is the total embodied CO_2_ of 1 m^3^ of foam concrete (in kgCO_2_−eq/m^3^).

**Figure 22:**
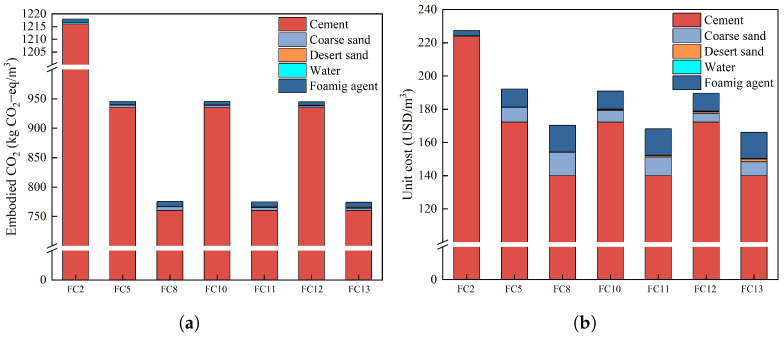
Comparison of embodied carbon emissions and unit costs of different foam concrete mixes. (**a**) Embodied carbon emissions breakdown. (**b**) Unit cost breakdown.

The embodied CO_2_ emissions, embodied energy, and cost results of DSFC and conventional foam concrete (FC) are presented in Figure 22 and Table 6. Analysis of the experimental data reveals that both the embodied CO_2_ emissions and the unit cost of foam concrete decrease with increasing sand-to-binder ratio (S/B), as evidenced by the comparison among mix proportions FC2, FC5, and FC8. Furthermore, when the sand-to-binder ratio is held constant, an increase in the desert sand replacement rate likewise leads to a reduction in embodied CO_2_ emissions and unit cost. However, owing to the relatively small differences in embodied CO_2_ emissions and unit cost between aeolian sand and river sand, the marginal impact of desert sand substitution on the overall embodied CO_2_ emissions and unit cost of the foam concrete remains limited.

Specifically, as shown in Table 6, the total embodied CO_2_ emissions of DSFC (FC13) and conventional FC (FC2) are 774.24 kgCO_2_−eq/m^3^ and 1218.00 kgCO_2_−eq/m^3^, respectively, indicating a 36.4% reduction in carbon footprint for DSFC compared with conventional FC. The principal driver of this reduction is the decrease in cement consumption in DSFC; given that cement is the dominant source of CO_2_ emissions in foam concrete systems, the embodied CO_2_ emissions associated with cement in DSFC (FC13) decreased by 37.5% compared with FC2, contributing more than 98% of the total carbon emission reduction. Notably, the embodied CO_2_ emission factor of desert sand is only 0.008 kgCO_2_−eq/kg, equivalent to 61.5% of that of standard sand, which further reinforces the low-carbon advantage of DSFC without introducing additional environmental burdens. Although the CO_2_ emissions from the foaming agent in FC13 (8.41 kgCO_2_−eq/m^3^) are higher than those in FC2 (1.47 kgCO_2_−eq/m^3^), its contribution to the total emissions is only 1.09% owing to the relatively small absolute dosage, exerting no significant negative impact on the overall low-carbon performance of DSFC. In terms of embodied energy, the total embodied energy of DSFC is 4897.40 MJ/m^3^, corresponding to a 34.1% reduction relative to conventional FC (7427.19 MJ/m^3^); this is consistent with the trend in CO_2_ emissions and further confirms the environmental benefits of DSFC in reducing life-cycle primary energy consumption.

From an economic perspective, the total cost of DSFC (FC13) is 166.14 USD/m^3^, corresponding to a 26.9% decrease compared with conventional FC (FC2 at 227.30 USD/m^3^) (Figure 22b). The principal reason for this cost reduction lies in the significant decrease in cement consumption; in addition, the unit cost of aeolian sand is only 28.6% of that of coarse sand, further lowering the aggregate cost of DSFC. Although the absolute cost of the foaming agent in DSFC is higher than that in conventional FC, its proportion in the total cost is only 9.52%, insufficient to offset the substantial cost reduction achieved by optimizing the cement and aggregate systems. Based on the environmental and economic assessments, the eco-strength efficiency (ESE) was further calculated using Equation (4). The results show that the ESE values of FC2 and FC13 are 44.45 kgCO_2_−eq/(m^3^·MPa) and 34.77 kgCO_2_−eq/(m^3^·MPa), respectively. Compared with FC, the ESE of DSFC is reduced by 21.8%, indicating a significant decrease in the carbon emission cost per unit of compressive strength.

**Figure 23:**
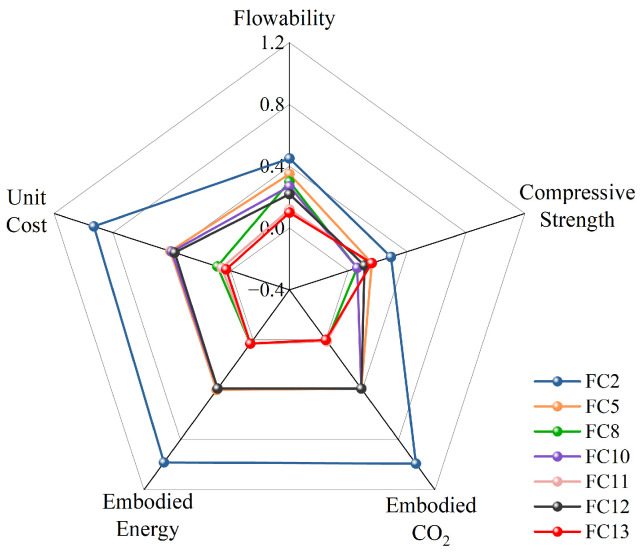
Multidimensional comparison of FC with different mix proportions.

To comprehensively evaluate the performance of DSFC, Figure 23 presents a radar chart comparing the flowability, compressive strength, embodied CO_2_ emissions, total embodied energy, and unit cost of seven foam concrete mix proportions. The chart indicates that increasing the sand-to-binder ratio and the desert sand replacement rate leads to a certain decline in flowability and compressive strength but simultaneously results in substantial reductions in embodied CO_2_ emissions and unit cost. For instance, compared with FC2, FC13 exhibits a 28.8% decrease in flowability and an 18.7% decrease in compressive strength (with a 28-day compressive strength of 22.3 MPa for FC13), while its CO_2_ emissions and unit cost decrease by 36.4% and 26.9%, respectively, leading to a 21.8% reduction in ESE.

In summary, compared with conventional foam concrete, DSFC with a high sand-to-binder ratio and a high aeolian sand replacement rate demonstrates significant advantages in low-carbon performance, eco-strength efficiency, and cost-effectiveness. This material system not only achieves the high-value utilization of aeolian sand as a solid waste resource but also provides a feasible technical pathway for the low-carbon transformation of concrete materials in the construction industry, offering promising potential as a strategic alternative for green buildings, low-carbon infrastructure, and ecological engineering projects.

## 4. Conclusions

In this study, the effects of the water-to-binder ratio (W/B), sand-to-binder ratio (S/B), and desert sand replacement rate on the macroscopic mechanical properties and microstructure of foam concrete were systematically investigated. Through experimental investigation, the intrinsic linkages among material composition, microstructural evolution, and macroscopic performance were elucidated. The principal conclusions can be summarized as follows.

1. The flow behavior of fresh DSFC is governed by the W/B ratio and solid phase particle size distribution. Increasing W/B from 0.3 to 0.5 improves flowability, with values reaching 160–200 mm only at W/B = 0.5. Conversely, raising S/B from 0 to 0.6 and desert sand replacement rate from 0% to 40% (at fixed W/B = 0.4) monotonically decreases flowability, driven by water adsorption and packing effects of fine desert sand. This competition between free water lubrication and the “water anchoring” effect from fine aggregates’ higher specific surface area reveals the rheological control mechanism unique to fine-aggregate-dominated cementitious systems.
2. The strengthening effect of desert sand on the macroscopic compressive strength exhibits a distinctive “S/B ratio dependence”, the essence of which lies in the dynamic competition between the “interfacial defect effect” under low S/B ratio conditions and the “fine aggregate filling effect” under high S/B ratio conditions. Specifically, at S/B = 0.6 and a 40% desert sand replacement rate, the compressive strength increases by 51.4% (from 14.4 MPa to 21.8 MPa) compared to the control, breaking the traditional perception that aeolian sand inevitably weakens the matrix.
3. A quantitative correlation linking the microscopic pore structure to the macroscopic mechanical properties was established, identifying D90 as a sensitive descriptor: compressive strength decreased by 50.8% when D90 increased from 270 µm to 330 µm, and by an additional 33.2% from 330 µm to 500 µm, with the strength decline rate plateauing beyond 500 µm. Microstructural analysis revealed that the dilution of the cementitious phase—reflected in the relative depletion of C-S-H gel and accumulation of weakly bonded portlandite—was the microscopic root cause of pore coarsening and macroscopic strength degradation, clarifying the cross-scale failure mechanism of DSFC.
4. Across the entire life cycle, the DSFC system exhibits green and low-carbon performance and engineering-economic feasibility. The optimization strategy combining an S/B ratio of 0.6 with a 40% desert sand replacement rate reduces the consumption of high-carbon cement clinker by 37.5% and lowers the eco-strength efficiency (ESE) by 21.8% relative to conventional foam concrete, demonstrating the substantive contribution of DSFC to minimizing the environmental cost per unit of strength.

In conclusion, the traditional dependence of foam concrete on high-quality river sand and high cement content is overcome. Theoretically, the synergistic and competitive mechanisms of the filler–interface duality in aeolian-sand-based composite cementitious systems are elucidated; practically, a green mix-design paradigm of “replacing cement with sand” and “filling coarse with fine” is established. These findings provide a scientific basis and a replicable technical model for the large-scale utilization of aeolian sand and the low-carbon transformation of building materials in arid, sand-scarce regions such as those surrounding the Taklamakan Desert.

## Figures and Tables

**Table 1:** Chemical compositions of ordinary Portland cement (OPC) (% by mass).

	SiO_2_	Al_2_O_3_	MgO	P_2_O_5_	K_2_O	SO_3_	CaO	Fe_2_O_3_	TiO_2_
OPC	25.94	6.86	1.82	0.12	1.06	4.69	53.35	4.87	0.37

**Table 2:** Chemical composition of desert sand (% by mass).

	SiO_2_	Al_2_O_3_	MgO	P_2_O_5_	Na_2_O	K_2_O	SO_3_	CaO	Fe_2_O_3_	TiO_2_	Cl
Desert Sand	51.39	6.48	2.10	0.07	1.18	1.81	8.01	21.06	6.69	0.65	0.36

**Table 3:** Relative densities of raw materials (kg/m^3^).

Materials	OPC	Standard Sand	Desert Sand	Foam Agent	Foam
Relative density	3180	2690	2610	1053	40

**Table 4:** Mix Design of Desert Sand Foam Concrete (kg/m^3^).

No.	Desert Sand (%)	S/B	W/B	Cement	Coarse Sand	Desert Sand	Water	Foam (L)
1	0%	0.0	0.3	1476.92	0	0	443.08	4.51
2	0%	0.0	0.4	1371.43	0	0	548.57	8.52
3	0%	0.0	0.5	1280.00	0	0	640.00	12.14
4	0%	0.3	0.3	1136.84	341.05	0	341.05	6.85
5	0%	0.3	0.4	1025.64	307.69	0	410.26	11.01
6	0%	0.3	0.5	930.77	279.23	0	465.38	14.74
7	0%	0.6	0.3	923.08	553.85	0	276.92	8.82
8	0%	0.6	0.4	833.33	500.00	0	333.33	12.96
9	0%	0.6	0.5	757.89	454.74	0	378.95	16.72
10	20%	0.3	0.4	1025.64	246.15	61.54	410.26	10.95
11	20%	0.6	0.4	833.33	400.00	100.00	333.33	16.05
12	40%	0.3	0.4	1025.64	184.62	123.08	410.26	10.89
13	40%	0.6	0.4	833.33	300.00	200.00	333.33	15.95

**Table 5:** Embodied CO_2_ and embodied energy values of various substances.

Materials	Mix Design (kg/m^3^)		Embodied CO_2_ Factor (kg CO_2_−eq/kg)	Embodied Energy Factor (MJ/kg)	Unit Cost (USD/kg)
	FC (FC2)	DSFC (FC13)			
Cement	1333.33	833.33	0.912 [29]	5.5 [29]	0.168
Desert sand	–	200	0.008 [30]	0.08 [30]	0.008
Coarse sand	–	300	0.013 [31]	0.11 [31]	0.028
Water	533.33	333.33	0.001 [29]	0.1 [29]	0.001
Foaming agent	2.79	15.95	0.527 [32]	14.53 [32]	0.991

**Table 6:** The embodied CO_2_ emissions, embodied energy values and cost results from DSFC and FC.

Materials	Embodied CO_2_ (kg CO_2_−eq/m^3^)		Embodied Energy (MJ/m^3^)		Unit Cost (USD/m^3^)	
	FC	DSFC	FC	DSFC	FC	DSFC
Cement	1216.00	760.00	7333.32	4583.32	224.00	140.00
Desert sand	–	1.60	–	16.00	–	1.60
Coarse sand	–	3.90	–	33.00	–	8.40
Water	0.53	0.33	53.33	33.33	0.53	0.33
Foaming agent	1.47	8.41	40.54	231.75	2.76	15.81
Total	1218.00	774.24	7427.19	4897.40	227.30	166.14

## Data Availability

The data presented in this study are available on request from the corresponding author.

## Abbreviations

The following abbreviations are used in this manuscript:

DSFC	Desert Sand Foam Concrete
FC	Foam Concrete
ITZ	Interfacial Transition Zone
W/B	Water-to-Binder Ratio
S/B	Sand-to-Binder Ratio
C-S-H	Calcium Silicate Hydrate
XRD	X-ray Diffraction
SEM	Scanning Electron Microscopy
EDS	Energy Dispersive Spectroscopy
LCA	Life-Cycle Assessment
ESE	Eco-Strength Efficiency
